# Effect of CuO Nanoparticles on the Optical, Structural, and Electrical Properties in the PMMA/PVDF Nanocomposite

**DOI:** 10.3390/mi14061195

**Published:** 2023-06-04

**Authors:** Amira Ben Gouider Trabelsi, Ayman M. Mostafa, Fatemah H. Alkallas, W. B. Elsharkawy, Ameenah N. Al-Ahmadi, Hoda A. Ahmed, Sherif S. Nafee, Rami Adel Pashameah, Eman A. Mwafy

**Affiliations:** 1 Department of Physics, College of Science, Princess Nourah Bint Abdulrahman University, P.O. Box 84428, Riyadh 11671, Saudi Arabia; aatrabelsi@pnu.edu.sa (A.B.G.T.); fhalkallas@pnu.edu.sa (F.H.A.); 2 Spectroscopy Department, Physics Research Institute, National Research Centre, 33 El Bohouth St., Dokki, Giza 12622, Egypt; 3 Department of Physics, College of Science, Qassim University, P.O. Box 6644, Buraydah Almolaydah 51452, Saudi Arabia; 4 Physics Department, College of Science and Humanities, Prince Sattam Bin Abdulaziz University, Alkharj 11942, Saudi Arabia; drwafaa8@gmail.com; 5 Department of Physics, Faculty of Applied Science, Umm Al-Qura University, Makkah 24230, Saudi Arabia; anahmadi@uqu.edu.sa; 6 Department of Chemistry, Faculty of Science, Cairo University, Cairo 12613, Egypt; ahoda@sci.cu.edu.eg; 7 Physics Department, Faculty of Science, King Abdulaziz University, Jeddah 21589, Saudi Arabia; snafee@kau.edu.sa; 8 Department of Chemistry, Faculty of Applied Science, Umm Al-Qura University, Makkah al-Mukarramah 24382, Saudi Arabia; rapasha@uqu.edu.sa; 9 Physical Chemistry Department, Advanced Materials Technology and Mineral Resources Research Institute, National Research Centre, 33 El Bohouth St., Dokki, Giza 12622, Egypt; emanmwafynrc@gmail.com

**Keywords:** PMMA/PVDF, CuO NPs, FT-IR, optical properties, electrical properties

## Abstract

A polymeric nanocomposite film, composed of PMMA/PVDF and different amounts of CuO NPs, was successfully prepared using the casting method to enhance its electrical conductivity. Various techniques were employed to investigate their physicochemical properties. The addition of CuO NPs causes a noticeable difference in the intensities and locations of vibrational peaks in all bands, confirming the incorporation of CuO NPs inside the PVDF/PMMA. In addition, the broadening of the peak at 2θ = 20.6° becomes more intense with increasing amounts of CuO NPs, confirming the increase in the amorphous characteristic of PMMA/PVDF incorporated with CuO NPs in comparison with PMMA/PVDF. Furthermore, the image of the polymeric structure exhibits a smoother shape and interconnection of pore structure associated with spherical particles that agglomerate and give rise to a web-like organization that becomes a matrix. Increasing surface roughness is responsible for an increasing surface area. Moreover, the addition of CuO NPs in the PMMA/PVDF leads to a decrease in the energy band gap, and further increasing the additional amounts of CuO NPs causes the generation of localized states between the valence and conduction bands. Furthermore, the dielectric investigation shows an increase in the dielectric constant, dielectric loss, and electric conductivity, which may be an indication of an increase in the degree of disorder that confines the movement of charge carriers and demonstrates the creation of an interconnected percolating chain, enhancing its conductivity values compared with that without the incorporation of a matrix.

## 1. Introduction

Polymeric nanocomposites are promising materials that can be used in industry and in research. They offer considerable property benefits at far lower loadings than the structure of polymer composites with typical micron-scale fillers, resulting in decreased component weight and the capacity to simplify processing. Furthermore, their versatile properties may open up new applications for polymers. They find widespread application in a variety of fields, including the information industry, packaging, safety, energy, transportation, electromagnetic shielding, defense systems, sensors, and catalysis [1,2,3]. They may provide solutions to a variety of issues and obstacles that are encountered on a regular basis in the real world, which gives these materials tremendous potential for the future [4]. They are created using the design premise that larger sizes and more surface areas are associated with significantly increased reactivity [4]. 

On account of polymeric materials, polymethyl methacrylate (PMMA) is one of the polymers that have garnered a lot of interest due to its transparent and colorless properties. It has a long life span, excellent mechanical and chemical stability, and high wettability. However, it has a few drawbacks, including low thermal stability, restricted electrochemical stability, and low cyclic rate efficiency. For that reason, there has been a lot of research over the past few decades showing that incorporating even trace amounts of nanofillers into polymers can improve their properties without negatively impacting their processability [5,6]. Therefore, when the nanofiller incorporates, embeds, or decorates itself across a polymer matrix, the structure of the resulting nanocomposite can produce promising properties. The nanofiller can be made of metals such as copper, gold, platinum, zinc, nickel, and silver, which are utilized in the production of nanocomposites for a wide range of applications across a number of different sectors of industry. Especially, the physicochemical properties of fillers will change when their size is reduced to the nanoscale size, which will be beneficial in a variety of applications [5,6]. 

A metallic filler that was recently used as a filler in polymeric nanocomposites, copper oxide (CuO), has a monoclinic crystal structure with good electrical conduction, which is related to their potential physical properties [7,8]. Crystalline CuO nanoparticles (CuO NPs) have a relatively small energy band gap, which allows them to be exploited for both photocatalytic and photovoltaic activities [9]. Because of the physicochemical properties of the nanocomposites that include CuO NPs incorporated into them, such as their semiconducting characteristics, great chemical stability, low toxicity, and chemical and physical stability, copper oxide has solidified its position as a significant substance in technology [10,11,12]. Moreover, it has become a promising option for use in energy storage systems. In addition, supercapacitors, a supplementary device between batteries and regular capacitors, have received a lot of attentions because of their advantageous properties [13]. Furthermore, it has also generated excitement for its potential use in the fabrication of supercapacitors’ positive electrodes. By looking at a previous work related to the use of polymeric nanocomposites for optoelectronic applications, Katowah et al. [14] studied a polymer nanocomposite composed of a PMMA/CuO/carbon nanofiller that was used as an electrochemical sensor for Hg^2+^. Rabee et al. [15] investigated the physicochemical properties of PMMA/CuO NPs and found that optical parameters such as band-gap energy decreased with increasing CuO NPs concentration. Alghunaim et al. [16] studied a PMMA/polycarbonate (PC)/CuO NPs system and discovered that adding CuO NPs reduces the values of energy band gaps of PMMA/PC while increasing the dielectric parameters (loss and constant). Abdullah et al. [17] found that the presence of CuO NPs decreases the band-gap energy of PVA, which confirmed the creation of a new energy state. Subashini et al. [18] detected that the addition of CuO NPs to glutaric acid, acrylic acid, and ethylene glycol improved their thermal stability and had antibacterial and anticancer properties. Manjunath et al. [19] added different weight percentages of CuO NPs to PVA using a simple casting method and found that the addition of CuO NPs increased the amorphous character of PVA and lowered the band-gap energy.

In order to enhance the conductivity of polymeric nanocomposite structures, PMMA was mixed with polyvinylidene fluoride (PVDF) to obtain the superior efficiency of the electrolyte with higher electrochemical stability. This was carried out in an effort to overcome the shortcomings that are associated with electrolyte systems that are based on PMMA polymers. This was due to the presence of two distinct types of intermolecular interactions. The carbonyl groups of PMMA can form hydrogen bonds with the methylene groups of PVDF. The other form of interaction is a dipole–dipole one, and it takes place between the methylene groups of PMMA and the difluoromethylene groups of PVDF [20]. Based on the literature survey, Jeedi et al. [20] studied the electrical properties of polymer nanocomposites of PVDF/PMMA/AgNO_3_ and found that the ionic conductivity was enhanced by the addition of AgNO_3_. Alghamdi et al. [21] studied the electrical and thermal properties resulting from the incorporation of graphene into PVDF/PMMA and found that both thermal and electrical conductivities are improved by the addition of graphene. Gaabour et al. [22] investigated the optical, spectroscopic, and magnetic properties of PVDF/PMMA/Fe_3_O_4_ and discovered that the addition of Fe_3_O_4_ decreased the PVDF/PMMA band-gap energy. Moreover, saturation magnetization values, which display paramagnetic behavior, are close to 75 emu/g for pure Fe3O4, and they decrease with decreasing Fe_3_O_4_ content. 

Herein, a series of different percentages of CuO NPs (1, 3, 5, and 7 wt.%) were added for the first time to the polymeric composite matrix structure of the PMMA/PVDF blend to enhance its electrochemical stability. After that, their physiochemical properties are investigated to determine the proper ratio that gives the best properties to be used in batteries.

## 2. Experimental Work

### 2.1. Materials

PMMA (M. wt. = 2,480,000) and PVDF (M. wt. = 534,000) were supplied by the ACROS company. CuO NPs were provided by Aldrich with a particle size of <50 nm. Dimethyl formamide (DMF) was purchased from SD Fine Chemicals.

### 2.2. Method of Preparation of PMMA/PVDF/CuO NPs

**Scheme 1:**
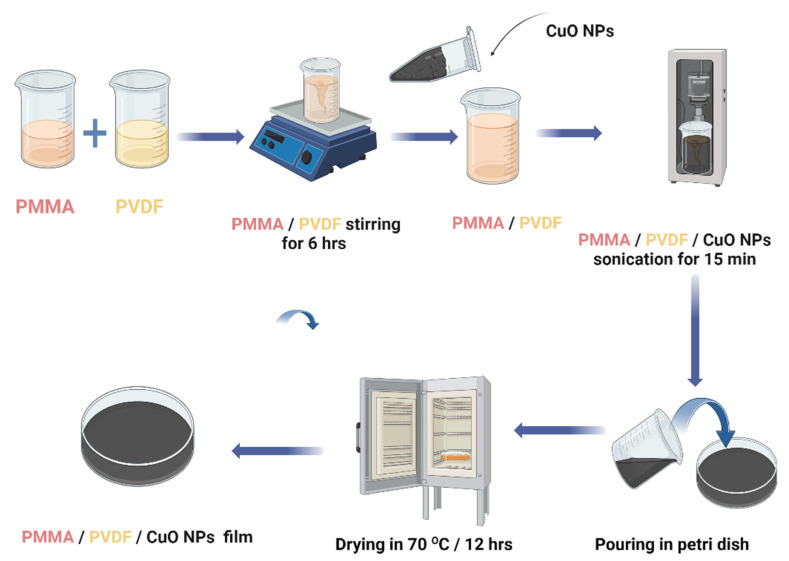
Experimental illustration of preparation steps of PMMA/PVDF/CuO NPs.

An equal amount of PMMA and PVDF (50/50 wt.%) was individually dissolved in DMF at 70 °C. After that, the two polymers were added to each other under continuous stirring to reach a homogenous blend solution. CuO NPs were inserted to the blend solution in the following mass ratios: 1, 3, 5, and 7 wt.%. The polymer nanocomposite solution was sonicated very well to avoid the agglomeration of nanoparticles. The resulting solution was transferred into Petri dishes and dried at 70 °C as described in Scheme 1.

### 2.3. Measurements Tools

Fourier transform infrared spectroscopy (FT-IR, VERTEX 80 spectrometer) and X-ray diffraction (Panalytical X’Pert PRO diffractometer) were utilized in order to carry out structural characterization on the samples that had previously been manufactured. In order to investigate the surface morphology, scanning electron microscopy (SEM, Quanta 250 FEG) was utilized. In order to verify the size and shape, a transmission electron microscopy (TEM, JEM-2100F) was utilized. The UV–visible spectrophotometry (UV–Vis, JASCO, V-630) method was utilized to know the optical characteristics. To determine AC conductivity, a broadband dielectric spectroscopy was used. In a parallel plate capacitor design, the measurements were taken with stainless-steel electrodes that were gold-plated on both sides.

## 3. Results and Discussions

**Figure 1:**
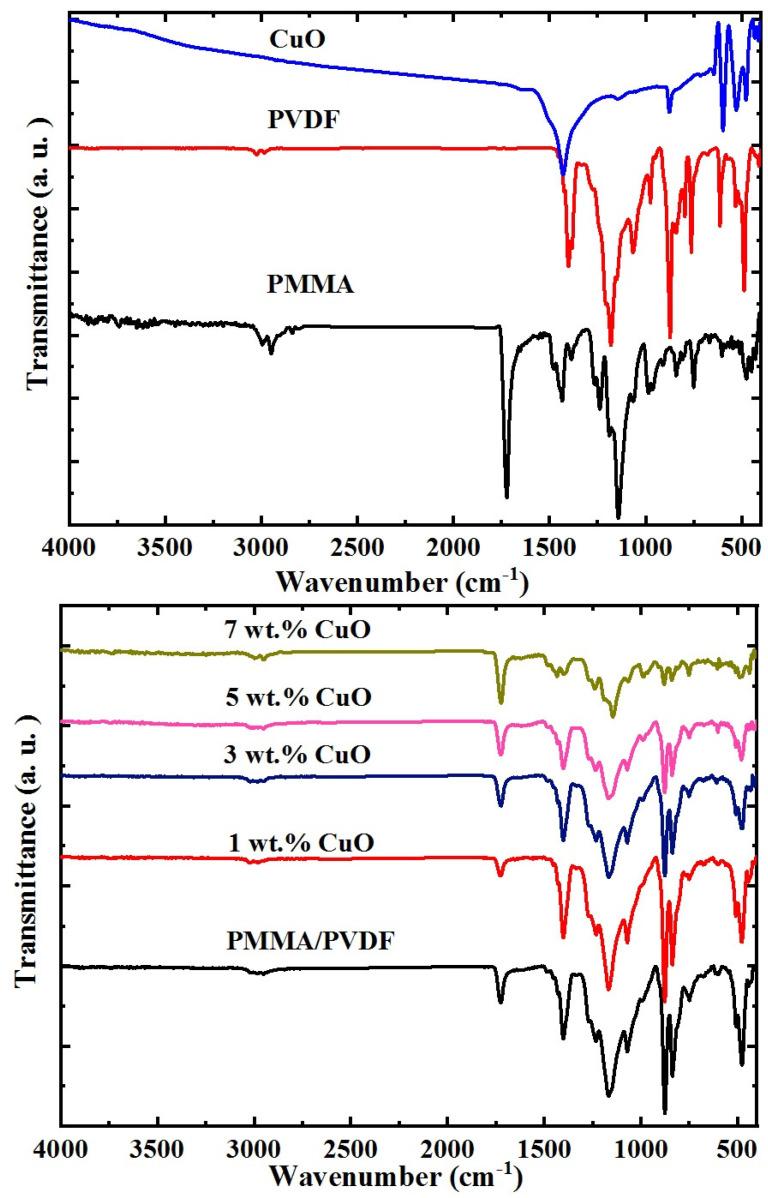
FT-IR of PMMA, PVDF, CuO, PMMA/PVDF, and PMMA/PVDF incorporated with different weight percentages of CuO.

Figure 1 represents the transmittance spectral analysis of PMMA, PVDF, CuO, PMMA/PVDF, and PMMA/PVDF incorporated with different amounts of CuO NPs. In the case of PMMA, the two characteristic stretching vibrational peaks located at 2998 and 2946 cm^−1^ are based on C–H functional groups. Furthermore, the intense stretching vibrational band that appeared at 1719 cm^−1^ is based on the C=O functional group. In addition, the bending vibrational peaks located at 1388 and 1435 cm^−1^ are related to the CH_3_ functional group. The stretching vibrational peaks that appeared around 1064 and 1142 cm^−1^ are based on the functional groups of C–O–C and C–O, respectively. The bending vibrational peak observed at 750 cm^−1^ is related to the C–H functional group [23,24,25,26,27]. In the case of PVDF, it has bands at 1405 and 1388 cm^−1^, which correspond to the bending vibration of C–H. Moreover, the stretching vibration peak that appeared at 1182 cm^−1^ is because of CF_2_. Furthermore, the wagging vibration peaks located at 1066 and 874 cm^−1^ are because of the C–H functional group. Moreover, the bending vibration peaks located at 841 and 792 cm^−1^ are based on the C–F functional group. In addition, the appearance of stretching vibrational peaks appearing at 530, 510, and 487 cm^−1^ are related to CF_2_ and the deformation of CF_2_ [28,29,30]. For the CuO NPs spectrum, the stretching vibrational peaks of Cu–O are showed at 528 and 432 cm^−1^. The wagging vibration of Cu–O is seen at 597 cm^−1^ [31]. In PVDF/PMMA, there is a considerable change in the intensities of all bands. In addition, bands at 1435 and 1388 cm^−1^ in PMMA have disappeared. Furthermore, there is a shift in the band of PMMA from 1142 to 1167 cm^−1^. The band at 792 cm^−1^ in PVDF has disappeared. Moreover, there is a shift in peaks at 530 and 487 cm^−1^ of PVDF to lower frequencies. The above results confirmed the complexation and miscibility between PVDF and PMMA. For PVDF/PMMA doped with different amounts of CuO NPs, a vibrational peak of Cu–O at 432 cm^−1^ appeared at the highest amount of CuO NPs. In addition, there is a shift in the band from 1167 cm^−1^ to a lower frequency of 1141 cm^−1^. As seen, the addition of CuO NPs causes a noticeable difference in the intensities of all bands, confirming the incorporation of CuO NPs inside the PVDF/PMMA blend and causing a structural change. 

**Figure 2:**
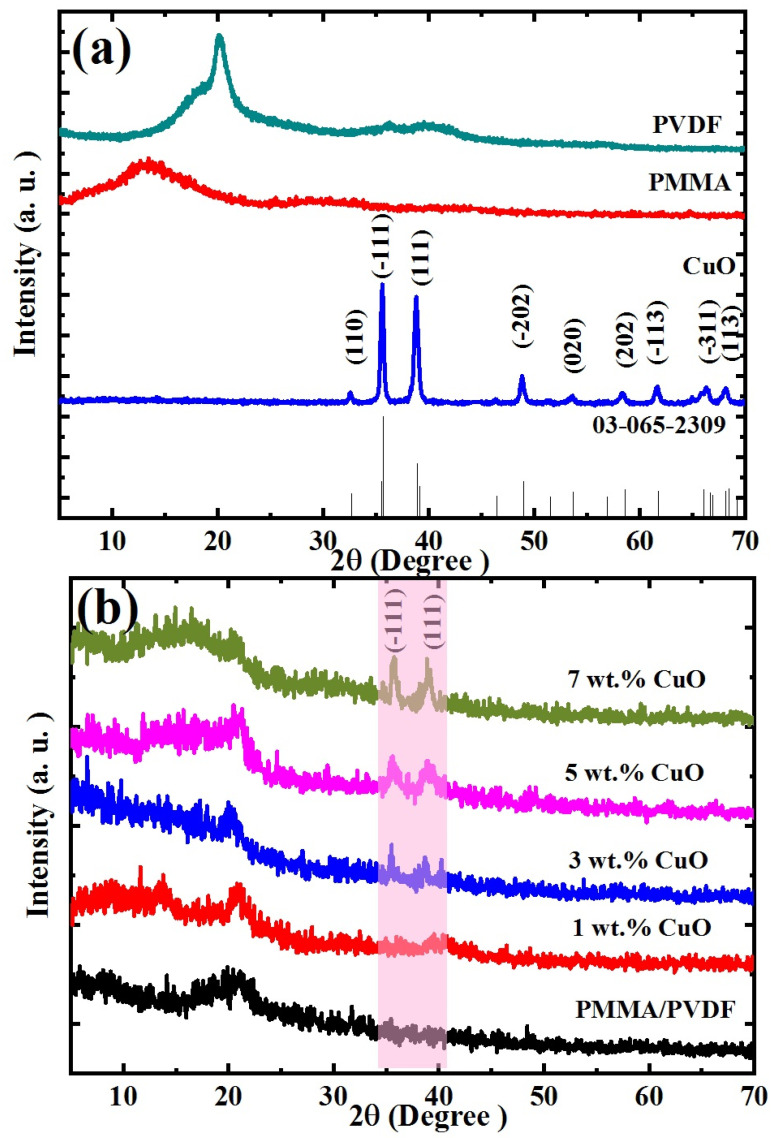
XRD of (**a**) PMMA, PVDF, CuO, PMMA/PVDF, and (**b**) PMMA/PVDF incorporated with different weight percentages of CuO.

Figure 2a represents an XRD analysis of PMMA, PVDF, and CuO. Diffraction patterns of CuO NPs can be observed at 2θ = 32.56° (110), 35.6° (002), 38.8° (111), 48.8° (−202), 53.6° (020), 58.2° (202), 61.6° (−113), 66.2° (−311), and 68.1° (113) according to card number (00-065-2309), which corresponds to the monoclinic phase of CuO NPs [32]. Scherrer’s equation [33] is used to make an estimation of the crystallite size of CuO. According to the investigation’s findings, the measured crystallite size of CuO NPs is 52 nm.

The absence of previously mentioned diffraction peaks and appearance of a broad peak at 13.3º give credence to the hypothesis that the PMMA polymer has an amorphous structure [34,35], while in the case of PVDF, it has a semicrystalline nature and diffraction peaks around 2θ values of 17.8°, 20.1°, and 36.2° [36,37]. For the PMMA/PVDF blend as observed in Figure 2b, there is a very broad diffraction peak at 2θ = 20.6° indicating that PMMA is able to engage with PVDF chains both in amorphous areas and at interphases to prevent PVDF segments from diffusing toward crystallization fronts, which suggests that PMMA is able to connect with PVDF chains [38]. After doping with CuO NPs, the diffraction peak at 2θ = 20.6° was shifted toward a higher value. Furthermore, with raising the weight percentage of CuO NPs, the two diffraction peaks stated to be observed at 2θ = 35.8° (−111) and 39.0° (111), which are related to the incorporation of CuO NPs in the matrix, and their intensities raising with increasing the amount of CuO NPs because of the successful interaction between the PMMA/PVDF structure and CuO NPs. The broadening of the peak at 2θ = 20.6° increases with raised CuO NPs amounts, confirming the increase in the amorphous character of PMMA/PVDF. As a result, the electrical conductivity of the nanocomposite system increases, as observed later.

**Figure 3:**
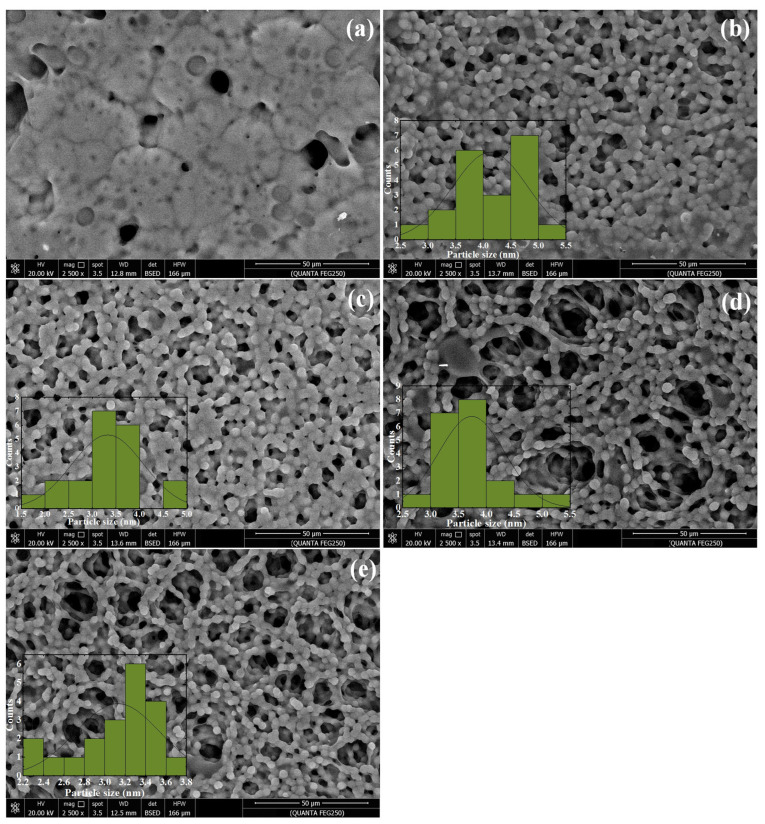
FESEM images of (**a**) PMMA/PVDF and PMMA/PVDF incorporated with (**b**) 1, (**c**) 3, (**d**) 5, and (**e**) 7 wt.% CuO.

Figure 3 shows FESEM images of PMMA/PVDF with and without the incorporation of different amounts of CuO NPs. From Figure 3a, the surface of the blended polymer films exhibits a smoother shape and an interconnected increase in the number of pores. This proves that amorphous properties predominate after semicrystalline ones are diminished, and this agrees with Mettu et al. [39]. Results from FESEM and XRD analyses both support the hypothesis that the material is amorphous. The surface morphological characteristics of PMMA/PVDF embedded with CuO NPs at different amounts are depicted in Figure 3b–e. As noticed, the surface of polymer nanocomposites reveals that they have a porous morphology. This occurs when spherical particles agglomerate and give rise to a web-like organization. It would appear that the typical particle size of these spherical particles is approximately 1.5–6 μm. Small pores provide a large surface area, which contributes to the material’s high binding capacity.

**Figure 4:**
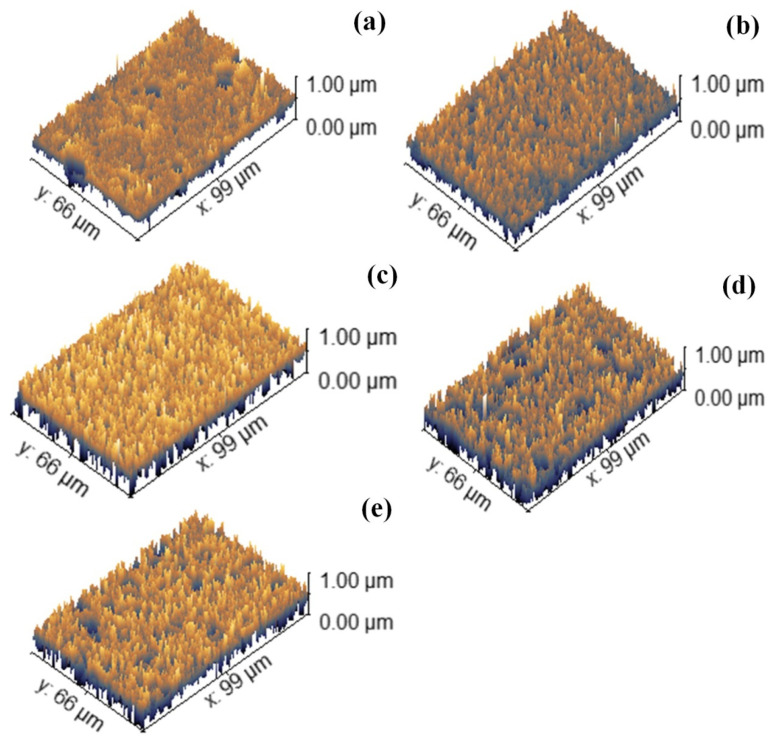
3D images of (**a**) PMMA/PVDF and PMMA/PVDF incorporated with (**b**) 1, (**c**) 3, (**d**) 5, and (**e**) 7 wt.% CuO.

The surface roughness is studied in Figure 4 using 3D images (Gwyddion software). Root mean square roughness (RMS) and roughness average (R_a_) are two measures used to characterize surface roughness [40]. The generated films’ physical adhesion is significantly affected by these characteristics. As compared with the pure PMMA/PVDF film as observed in Table 1, the addition of CuO NPs to the polymer mixture results in increasing surface roughness, which is responsible for an increasing surface area.

**Figure 5:**
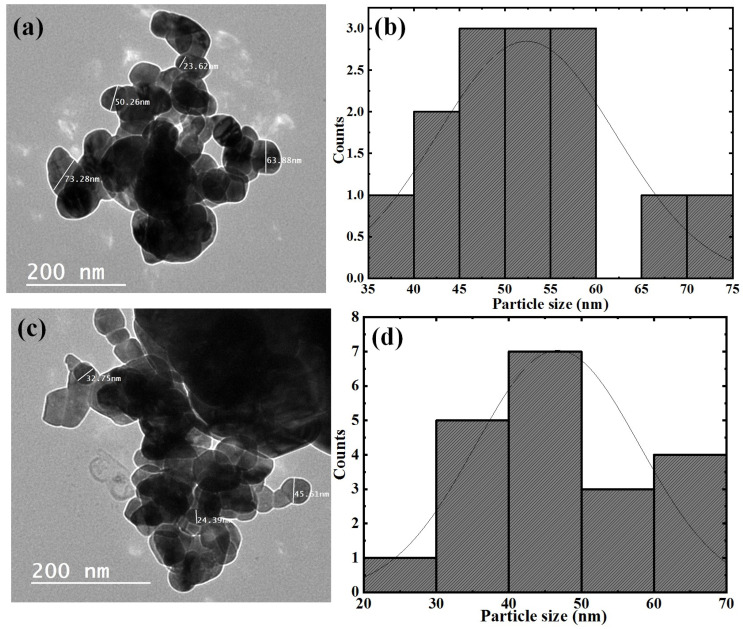
(**a**,**b**) HRTEM and (**c**,**d**) histogram of CuO NPs.

Figure 5a,b represent the HRTEM of CuO NPs. As observed, CuO NPs have a semispherical shape, and the particles appear to have agglomerated with each other. Figure 5c,d represent the histogram of CuO NPs using ImageJ software (open access). The average particle size of CuO NPs is around 53 nm.

**Figure 6:**
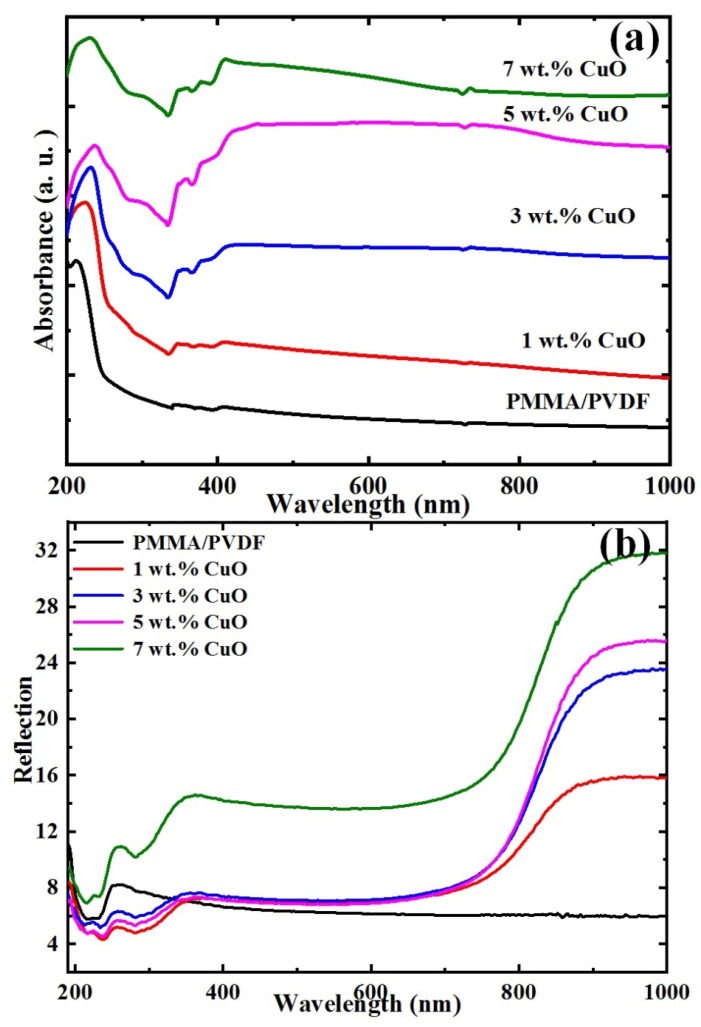
(**a**) Absorbance and (**b**) diffuse reflectance of PMMA/PVDF and PMMA/PVDF incorporated with CuO NPs.

Figure 6a show the absorption spectra of PMMA/PVDF with and without the incorporation of different amounts of CuO NPs. Pure PMMA/PVDF has an absorption peak at 213 nm based on n-π* transition [22]. As the weight percentage of CuO NPs rises, the absorption peak at 213 nm is red-shifted. In addition, the peak broadening increases with increasing CuO NPs. Moreover, there is a new appearance of an absorption peak at 350 nm with the addition of CuO NPs [41]. These findings demonstrate that the interaction of the CuO NPs with the functional groups in the PMMA/PVDF leads to a change in the energy band gap.

Figure 6b shows the diffuse reflectance (R) of PMMA/PVDF with and without the incorporation of different amounts of CuO NPs. It is observed that the reflectance of all samples increases at 𝜆 ≥ 400 nm. It is worth noting that increasing the CuO NPs content in PMMA/PVDF polymers increases the value of reflectivity. When the reflectance is high, it means that light cannot get through thin-film samples in a significant way. 

Absorption spectra changes can be utilized to predict their electron transitions. The basic absorption associated with band-to-band or exciting transitions is characterized by a significant increase in absorption, which is referred to as the optical band-gap energy (E_g_) [34]. Absorption can be expressed as a function of the medium’s length using an absorption coefficient (α), which was used to calculate (α) using Beer–Lambert’s relation [34].

**Figure 7:**
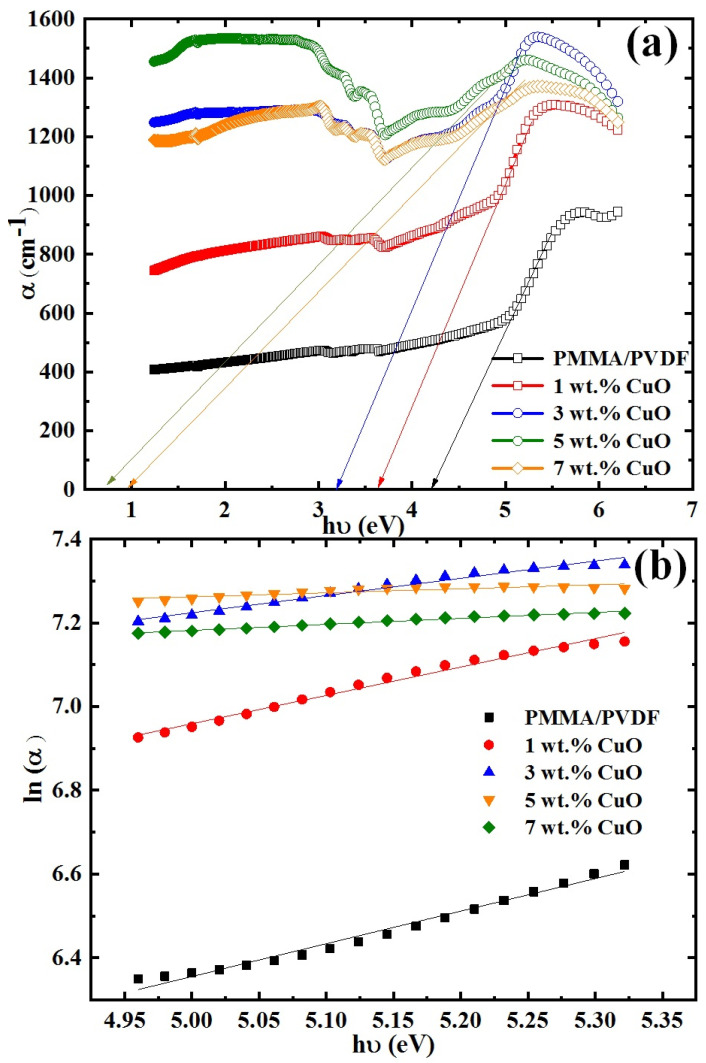
Relation between (**a**) α and (**b**) lnα versus photon energy (h𝜐) of PMMA/PVDF and PMMA/PVDF incorporated with CuO NPs.

The relationship between α and h𝜐 is seen in Figure 7a. The computed values of the absorption edge are determined by extrapolating the linear component till zero X-axis. It is clear from Table 2 that the presence of CuO NPs causes the absorption edge of PMMA/PVDF to move in the direction of low photon energy. This can be seen by comparing the value of 4.2 eV for PMMA/PVDF with the value of 1.0 eV for the PMMA/PVDF/7 wt.% CuO NPs sample. The decrease in the values of the absorption edge was generated by the addition of CuO NPs is reflective of the modifications that occurred in the sample band structure. These changes might be the consequence of the formation of new localized states in the band gap [42]. This would suggest that the incorporation of CuO NPs into the PMMA/PVDF matrix brings about a decrease in the band-gap energy.

The Urbach tail is an exponential portion of the absorption coefficient curve that lies along the curve and close to the absorption edge. Because disordered and amorphous materials have localized states that extend within the usual band gap, these materials exhibit an exponential tail in their electronic band structure [43]. The empirical rule of Urbach is demonstrated by studying the absorption coefficient (α) versus h𝜐 in this range [43].

From Figure 7b, the Urbach energy value (E_u_) can be estimated by extrapolating straight line till X-aix zero value that represents the Urbach plot (lnα against h𝜐) for each of the processed samples. Because of the amorphous character of the system, the best results of the straight-line fitting show that the current PMMA/PVDF/CuO films satisfy the Urbach empirical rule. This is confirmed by the straight-line fitting. The slope reciprocal of the Urbach tail gives the values of E_u_. The creation of defects in the band structure is shown to be confirmed by the increase in E_u_ (as observed in Table 2) that occurs in response to an increase in CuO NPs concentration. This indicates that the amorphous state of the matrix has increased. As a result, the values of E_g_ reduce as well.

**Figure 8:**
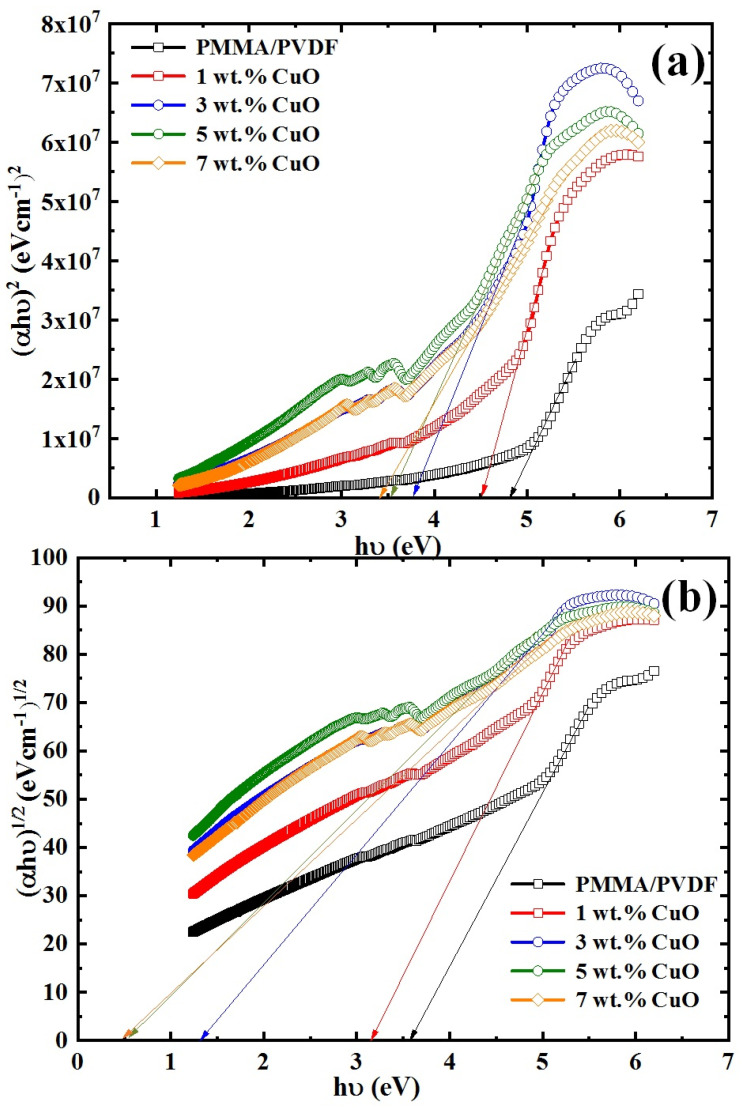
(**a**) Direct and (**b**) indirect transition of PMMA/PVDF with and without incorporation of CuO NPs.

Using Tauc’s plot, one can define the relation of α versus h𝜐 [44]. The values of direct (E_d_) and indirect (E_id_) band-gap energy are defined by extending the straight line to intercept the h𝜐-axis from the plot of (αhʋ)^2^ and (αhʋ)^½^ against h𝜐 as seen in Figure 8a and Figure 8b, respectively. It was discovered from Table 2 that the values of E_g_ are reduced as the CuO NPs content in PMMA/PVDF films increased, which based on the localized electron states was formed at the defect place in the band-gap structure depending on CuO NPs feeding on the PMMA/PVDF network structure. This conclusively demonstrates that the ion transfer between CuO NPs and the PMMA/PVDF matrix may be improved by using nanocomposites. An examination of the band tail width with the empirical Urbach equation provided further evidence that this result is accurate. This indicates that the incorporation of CuO NPs has a tendency to disperse the energy states, making it possible for a significant number of bands to transition into tail states and tail states to transition into other tail states, especially for 5 wt.% and 7 wt.% of CuO NPs. It is important to highlight the fact that the small amount variations of polymer composite with tunable E_g_ have different applications in photovoltaic and optoelectronic devices.

**Figure 9:**
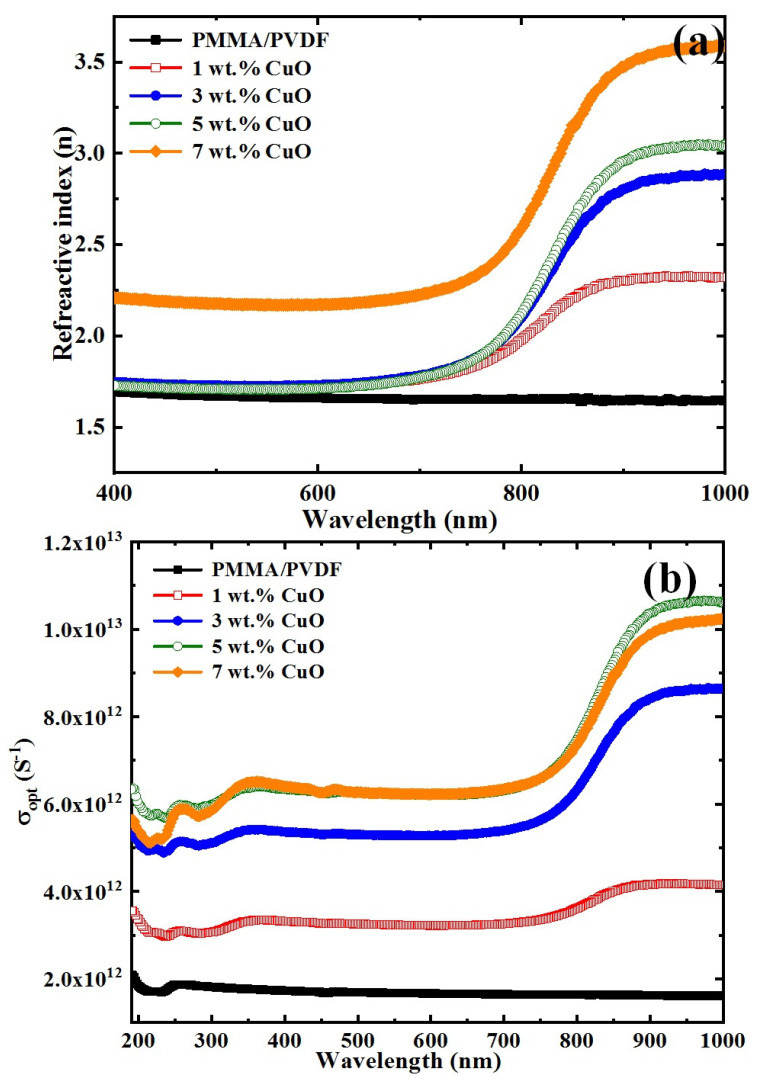
Relation between (**a**) n and (**b**) optical conductivity versus wavelength of PMMA/PVDF and PMMA/PVDF incorporated with CuO NPs.

In order to choose the appropriate materials for a particular optoelectronic application, it is necessary to do research on optical constants such as the refractive index. In addition, the optical properties are directly linked to electrical properties of the material. Figure 9a represents the change of n with wavelength. The value of n is calculated from the equation [45].

As observed in Figure 9a, the values of the refractive index increase with increasing wavelength and CuO NPs content. The higher the CuO NPs loading, the greater the number of charge carriers that are introduced into the host polymer. Therefore, there will be a higher density as well as a greater number of polarizable molecules, which will lead to an increased index of refraction. The evaluation of the optical materials’ refractive indices is important for applications in integrated optical devices, and it is a fundamental parameter for the design of the devices [46]. Therefore, the increase in n of the PMMA/PVDF/CuO NPs system demonstrates that it is suitable for a number of different applications.

In addition, the optical conductivity (σ_opt_), is an additional essential optical parameter because it elucidates the charge–transfer complex that exists between the filler CuO NPs and PMMA/PVDF polymeric mix. The optical conductivity of the produced films can be detected from the values of n, speed of light (C), and α [47].

As seen in Figure 9b, the values of optical conductivity increase with increasing CuO NPs content. This improvement in optical conductivity that occurs with a rise in CuO NPs is the result of an increase in both the concentration of charge carriers and the absorption of photons that are incident on the material.

The polymeric nanocomposites exhibit conduction and polarization effects via charge transport when subjected to an applied electric field. If an electric field is introduced to one of these nanocomposites, the charges will shift about locally, creating what are called induced dipole moments. Strong linkages exist between the phenomenon of polarization and the electrical characteristics of dielectric composites. When an electric field is applied to a material, the dielectric permittivity (ε* = ε′ + iε″) of the material can be utilized as a physical property to identify the level of polarization [48].

**Figure 10:**
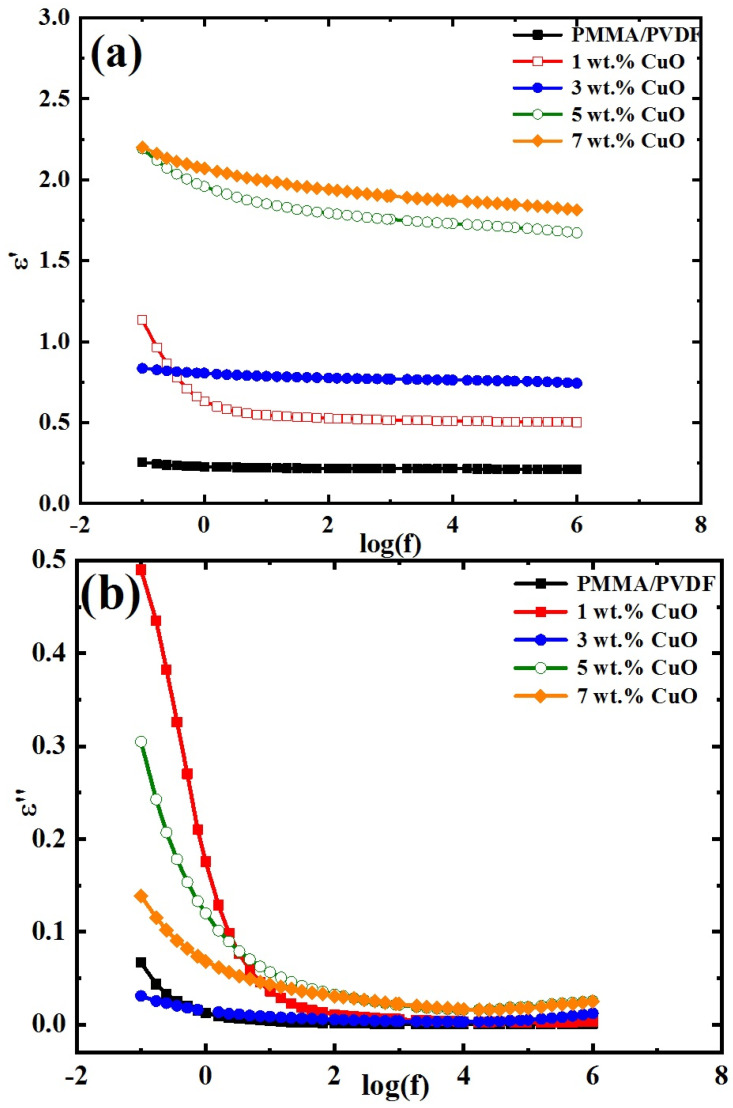
Relation between dielectric parameters: (**a**) constant and (**b**) loss versus frequency of PMMA/PVDF and PMMA/PVDF incorporated with CuO NPs.

ε′ is the sample’s standard dielectric constant, and ε″ refers to the dielectric loss. Figure 10a represents the relation between ε′ and frequency. Because the values of ε′ drop with increasing frequency, the dipole is difficult to accurately and easily spin, and its oscillation begins to occur after this field. This is because the ε′ values decrease. In addition, there was no evidence of relaxation peaks, which points to a non-Debye response. Figure 10b represents the relation between ε″ versus frequency, which shows that the motion of ions is recognized as the fundamental foundation of nanocomposite ε″ at lower frequencies, leading to a decrease in ε″ as a function of rising frequency. Thus, the high value of the dielectric loss at low frequencies indicates the impact of ion jumping and the loss in ion for both of movement conduction and polarization. However, the main source of ε″ is the vibrations of ions, which occur at a higher frequency and thereby reduce ε″ [48].

Figure 10 shows that due to the significantly higher values of ε′ and ε″ for the filled films, they have a greater ε″ than the PMMA/PVDF polymer matrix. This increase is indicative of an increase in the effective parallel ordering of dipoles as a result of electrostatic interactions between the PMMA/PVDF functional groups and CuO NPs, which would explain why the dielectric polarization of incorporation samples has increased. Additionally, the doped films increased energy loss per cycle, which is represented by the high values of ε″. 

**Figure 11:**
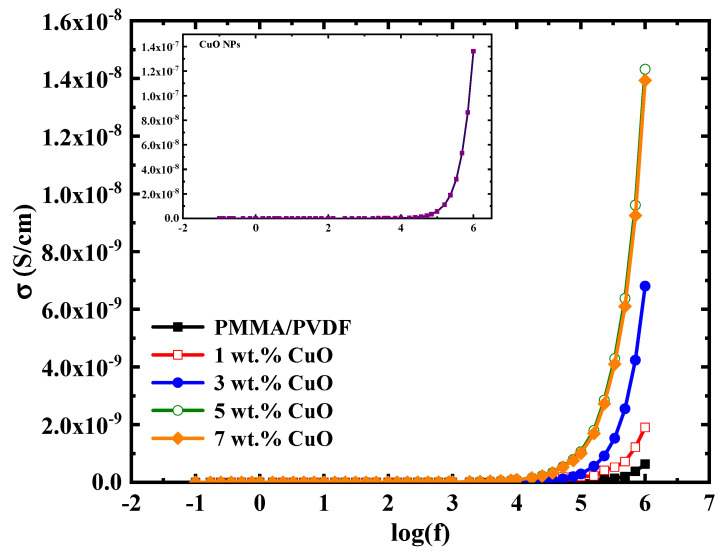
Effect on conductivity versus frequency of CuO NPs, PMMA/PVDF, and PMMA/PVDF incorporated with CuO NPs.

The conductivity of CuO NPs and PMMA/PVDF with and without the incorporation of CuO NPs against frequency was investigated in Figure 11. At low-frequency levels, conductivity is poor due to interfacial impedance or space charge polarization. This indicates that the thin films that have been investigated have non-Debye properties. Conductivity increases with a rise in frequency when operating at higher frequencies. Furthermore, the conductivity values of PMMA/PVDF incorporated with CuO NPs observed higher values compared with those without incorporation, confirming that the incorporation process of CuO NPs in the polymeric structure of PMMA/PVDF improves the mechanism of charge conduction in a moderately faster approach, enhancing the amount of disorder, which limits the movement of charge carriers, and showing the construction of a linked percolating chain ideal for the charge transfer process. Manjunath et al. [19] studied the effect of CuO NPs on the electrical properties of PVA and concluded that the dielectric constant was (1.4 × 10^−1^) and conductivity was (5.5 × 10^−8^). Mohammed [49] investigated the conductivity of PMMA/PVDF/ZnO and found that, for higher concentrations of ZnO, the conductivity became (1 × 10^−11^). Zhang et al. [50] showed that the incorporation of graphite nanosheets into a PVDF polymer increases the conductivity to (1 × 10^−12^). In our work, the electrical conductivity reached (1.4 × 10^−8^).

## 4. Conclusions

In this study, PMMA/PVDF polymers loaded with CuO NPs were successfully produced by a casting technique. The complexation and interplay between PMMA/PVDF and CuO NPs were verified by FT-IR analysis. Findings from the X-ray diffraction study show that adding CuO NPs to a PMMA/PVDF blend alters the crystallinity level and makes the blend more amorphous. The addition of CuO NPs to the PMMA/PVDF blend altered its surface shape, as shown by FESEM, and also enhanced its roughness. As the nanofiller content was increased, the resulting films exhibited a general decrease in their absorption edge and band-gap energy values. The addition of CuO NPs revealed a rise in the charge carrier density inside the PMMA/PVDF blend, which was reflected in a rise in Urbach energy, refractive index, and optical conductivity. The incorporation of CuO NPs into the PMMA/PVDF chain led to an increase in both the AC conductivity and complex permittivity values.

## Figures and Tables

**Table 1:** Values of RMS and R_A_ of PMMA/PVDF incorporated with different amounts of CuO NPs.

CuO NPs (Wt.%)	RMS (nm)	R_A_ (nm)
0	115	86
1	132	104
3	173	131
5	177	147
7	183	151

**Table 2:** Absorption edge, Urbach energy, direct (E_d_), and indirect (E_id_) energy band gap of PMMA/PVDF incorporated with CuO NPs.

CuO wt.%	Absorption Edge (eV)	Urbach Energy	Energy Gap (eV)	
		(eV)	E_d_	E_id_
0	4.2	1.3	4.8	3.6
1	3.6	1.5	4.5	3.1
3	3.2	2.4	3.8	1.3
5	0.7	10.4	3.5	0.5
7	1.0	7.0	3.4	0.6

## Data Availability

Not applicable.
